# Potential Synergistic Action of Bioactive Compounds from Plant Extracts against Skin Infecting Microorganisms

**DOI:** 10.3390/ijms21145105

**Published:** 2020-07-19

**Authors:** Przemysław Sitarek, Anna Merecz-Sadowska, Tomasz Kowalczyk, Joanna Wieczfinska, Radosław Zajdel, Tomasz Śliwiński

**Affiliations:** 1 Department of Biology and Pharmaceutical Botany, Medical University of Lodz, 90-151 Lodz, Poland; 2 Department of Economic Informatics, University of Lodz, 90-214 Lodz, Poland; anna.merecz-sadowska@uni.lodz.pl (A.M.-S.); radoslaw.zajdel@uni.lodz.pl (R.Z.); 3 Department of Molecular Biotechnology and Genetics, University of Lodz, 90-237 Lodz, Poland; tomasz.kowalczyk@biol.uni.lodz.pl; 4 Department of Immunopathology, Medical University of Lodz, 90-752 Lodz, Poland; joanna.wieczfinska@umed.lodz.pl; 5 Laboratory of Medical Genetics, Faculty of Biology and Environmental Protection, University of Lodz, 90-236 Lodz, Poland; tomasz.sliwinski@biol.uni.lodz.pl

**Keywords:** plant extracts, immunological response, skin diseases, modulation of skin immunity, skin pathogens

## Abstract

The skin is an important organ that acts as a physical barrier to the outer environment. It is rich in immune cells such as keratinocytes, Langerhans cells, mast cells, and T cells, which provide the first line of defense mechanisms against numerous pathogens by activating both the innate and adaptive response. Cutaneous immunological processes may be stimulated or suppressed by numerous plant extracts via their immunomodulatory properties. Several plants are rich in bioactive molecules; many of these exert antimicrobial, antiviral, and antifungal effects. The present study describes the impact of plant extracts on the modulation of skin immunity, and their antimicrobial effects against selected skin invaders. Plant products remain valuable counterparts to modern pharmaceuticals and may be used to alleviate numerous skin disorders, including infected wounds, herpes, and tineas.

## 1. Introduction

Dermatological disorders are the fourth leading cause among nonfatal disability worldwide [1]. In family medicine, the prevalence of skin disease accounts for 12.4% of all disorders [2] and depends on sociodemographic circumstances [3]. Premature death associated with skin disorders is the 18th leading cause of health burden [4].

Bacteria, viruses, and fungi are common pathogens which affect skin. Fungal skin disease counts towards the top 10 most prevalent diseases globally, whereas bacterial (impetigo) and viral (molluscum contagiosum/warts) skin diseases are in the top fifteen [4]. The first line of defense is the epidermis, which acts as a physical barrier [5]. This is supported by the skin immune response, which reacts after wounding and infection [6]. The clinical manifestations and severity of skin infections varies according to disease.

Medicinal plants have been used in traditional medicine for several thousand years in virtually all cultures. About 200 years ago, our pharmacopoeia was dominated by herbal medicines [7] and about 25% of the natural compounds prescribed worldwide came from plants [8]. Historically, all medicinal preparations were derived from plants, whether as plant parts (leaf, rhizome, roots, stem, bark or fruits) or as crude extracts or mixtures. Plant extracts contain a number of bioactive compounds of different classes, including alkaloids, terpenoids, and polyphenols, possess numerous activities, such as antibacterial, antiviral, antifungal, antioxidant, anti-inflammatory, and anti-obesity [9,10,11,12,13,14], and have been used in various disorders, such as cardiovascular diseases, rheumatoid arthritis, osteoporosis, and skin diseases [15,16,17,18,19]. During the last few decades, the use of traditional medicine has expanded globally and has gained popularity as primary health care in developing countries, and in countries where conventional medicine is predominant [20].

Plant medicines also provide a rational means of treating many diseases that are incurable in other systems of medicine [21,22,23,24]. According to the World Health Organization (WHO), about 80% of the world’s population in developing countries depends essentially on plants for their primary healthcare and lack access to modern medicine. Additionally, of the 252 drugs considered as basic and essential by the WHO, 11% are exclusively of plant origin and a significant number are synthetic drugs obtained from natural precursors [25,26,27].

In dermatology, plants can have both beneficial and adverse effects on skin. The book “Botanical Dermatology” by Mitchell and Rook lists more than 10,000 species which cause irritative or allergic contact dermatitis [28]. Phytodermatitis is defined as inflammation of the skin caused by a plant. The clinical patterns may be allergic phytodermatitis, photophytodermatitis, irritant contact dermatitis, pharmacological injury, and mechanical injury [29,30]. The data supporting the use of phytotherapy are limited and therapies may not be regulated and standardized [31]. However, with the development of technology, it has become possible to determine the pharmacology and mechanisms of action of medical plants and they have already made fruitful contributions for modern medicine [20]. Moreover, about one-third of all traditional medicines are used to heal skin diseases, compared with just 1–3% of modern drugs [32,33,34,35]. A review of the literature published over the past five years indicates a number of plant extracts that demonstrate antibacterial, antifungal or antiviral activity against skin diseases.

The aim of this study was to evaluate the role of plant extracts in the modulation of the cutaneous immunological response. It also examines their activity against selected skin pathogens, including bacteria, viruses, and fungi, with a focus on the molecular mechanisms of their action against skin invaders.

## 2. Immunological Response of the Skin

The skin is the largest organ of the human body, and one which plays an important role in protecting the body from harmful exposure to external and internal environments. It is not only a physical barrier but also an immunological one. The cutaneous immune response involves keratinocytes and Langerhans cells (LCs) located in the outer layer, the epidermis, as well as mast cells residing in the dermis, and T cells in both layers [6]. They are responsible for innate and adaptive immunological reactions which promote cutaneous inflammation and memory response against antigens [36]. Innate immunity is a nonspecific rapid response, whose role is to activate phagocytes to neutralize microorganisms like bacteria, viruses, and fungi by phagocytosis [37]. In contrast, adaptive immunity is a long-lived process that requires interaction between T lymphocytes; these are subdivided into two groups, CD4 and CD8, depending on the presence of cell surface molecules and the cells presenting the antigens. This creates an immunological effector pathway specific for particular pathogens and initiation of the lymphocyte immunologic memory. These processes are crucial for maintaining immune homeostasis in the body and protect against harmful pathogens [38,39].

Keratinocytes, LCs, T lymphocytes, and mast cells express pathogen-recognition receptors (PRRs). Toll-like receptors (TLRs), members of that group, are involved in the host defense process against various pathogenic microorganisms. Thus far, thirteen TLRs have been identified in mammals. Each TLR detects different microbial components. TLR2/1 recognizes lipopeptides, TLRs 3/7/8/13 identify RNA, lipopolysaccharide (LPS) for TLR4, TLR5 recognize flagellin, TLR9 identify unmethylated CpG islands in DNA, profilin and *Salmonella* flagellin for TLR11, and profilin for TLR 12 [40,41]. Toll signaling pathways activate a wide range of genes via induction transcription factor NF-κB, AP-1, and interferon regulatory factors 3 and 7 (IRF3/7); these stimulate the expression of a variety of biologically active molecules. The most important secreted molecules are: tumor necrosis factor α (TNF); interleukins (IL) IL-1β, IL-6, IL-12 belonging to the cytokine family; IL-8, growth-regulated oncogene-α (GRO-α); monocyte chemoattractant protein (MCP) -1, -2, -3, -4; macrophage inflammatory protein-1 (MIP1) α/β; RANTES (regulated upon activation, normal T cell expressed and secreted) chemokine members; beta-defensins and cathelicidin antimicrobial agents; CD40, CD80 and CD86 co-stimulatory compounds; ICAM-1, which takes part in cell adhesion [42]. The adapter proteins include MyD88, Toll-interleukin 1 receptor domain-containing adapter protein (TIRAP), Toll-interleukin 1 receptor domain-containing adapter-inducing interferon-β (TRIF), and TRIF-related adapter molecule (TRAM). All TLRs, except TLR3, recruit MyD88. MyD88 initiates a downstream signaling cascade that results in the activation of the nuclear factor kappa-light-chain-enhancer of activated B cells (NF-κB), activator protein 1 (AP-1) and IRF3/7 and the expression of pro-inflammatory cytokines and antimicrobials. TIRAP and MyD88 are necessary for TLRs 2 and TLRs 4 initiation signaling of NFκB and mitogen-activated protein (MAP) kinases. TLR3 utilizes TRIF and especially stimulated IRF3/7, involved in the production of type I interferon (i.e., IFNα and IFNβ), which is important in the immune response against viruses. These mechanisms play an important role in the cutaneous immune response against numerous pathogens [40,42,43].

## 3. Modulation of Skin Immunity by Plant Extracts

Natural active compounds contained in plant extracts may impact on cutaneous immunological processes. Phytochemicals may act both as factors boosting immune response, but also as anti-inflammatory ones. Most of these studies were performed in vitro using the keratinocytes and mast cells. Keratinocytes are epidermal cells that act as structural elements [44]. Mast cells are on the upper dermal layer of the skin. Both play a role in skin immunity. Mast cells are activated by numerous factors, including microbial products. This is followed by degranulation and the release of various mediators, such as serine proteinases, histamine, cytokines, chemokines, and growth factors [45,46,47].

On the one hand, activation of skin immunity responses may make the immune system reactive and effective, accounting for the clinical prevention and treatment of skin disorders through direct application. A wide range of plants may be responsible. A study performed on human keratinocytes treated with bark of *Rauvolfia nukuhivensis* indicated that this extract inhibits keratinocyte hyperproliferation upon stimulation with IL-22 [48]. Another study also conducted on keratinocytes treated with arabinogalactan proteins derived from *Acacia senegal* and *Adansonia digitata* seeds showed upregulation of IL-1β genes, associated with inflammation [49]. An in vitro study on guinea pig skin showed that *Sinapis alba* L. (white mustard) administration is responsible for a reduction in LCs density and enhanced the release of IL-1 and TNF-α [50].

On the other hand, plant extracts have been shown to exert a beneficial role in inhibiting the activation of cells taking part in cutaneous immunological response. Several lines of evidence suggest that plant extracts are able to inhibit mast cells activation. Mast cells have secretary granules containing inflammatory mediators. Activation results in degranulation. Among plant extracts, *Vitex rotundifolia* leaf, *Prunus sargentii* leaf, *Lycoris aurea* underground part, *Hydrangea serrata* for acuminata aboveground part, *Prunus takesimensis* stem-bark, *Clintonia udensis* whole plant, and *Rhamnus davurica* leaf are the examples that significantly impact on degranulation and inhibit the process in mouse bone marrow-derived mast cells and RBL-2H3 cells. It has also been investigated that roots of *Sanguisorba officinalis* have the ability to degranulate IgE/Ag-activated mouse bone marrow-derived mast cells [51,52]. A study conducted on Wistar rat peritoneal mast cells suggests inhibition of histamine release upon treatment with *Schizonepeta tenuifolia* whole plant extract [53]. *Rhamnus davurica* leaf extract demonstrated the potent inhibitory effect on Fyn factors belonging to the Src family kinase that have a role in the activation of Syk followed by activation of downstream signaling cascades, leading to mast cell activation [54]. Moreover, it has been shown that phenol-rich plants such as leaves of *Camellia sinensis* and *Ocimum basilicumn* are able to stabilize mastocytes and thus, their antioxidant activity [55]. In keratinocytes, *Sanguisorba officinalis* roots suppressed chemokine production triggered by TNF-α and IFN-γ [51]. *Paeonia lactiflora Pallas* root extract acts as an inhibitor of expression crucial cytokines in keratinocytes, such as IL-6, IL-8, and TNF-α through downregulation of the NF-κB pathway [56].

Additionally, the dysregulation of skin immunity response may result in skin inflammatory diseases often related to abnormal expression of the mediators. In such cases, a plant extract with anti-inflammatory potential is needed. *Cannabis sativa* flower extract takes part in the downregulation of genes strictly related to inflammation and the mode of the NF-κB pathway in a human keratinocyte line [57]. The inhibitory effect of *Artemisia argyi* leaf extract on IFN-γ, TNF-α, IL-6, and IL-10 and immune cell infiltration on male Balb/c mice were assessed [58]. Another study was conducted on Swiss albino mice with induced edema. Two leaf extracts from *Kalanchoe brasiliensis* and *Kalanchoe pinnata* were administered topically and both of them had an inhibitory effect on myeloperoxidase activity, the levels of IL-1β, and TNF-α (pro-inflammatory cytokines) and increased the levels of IL-10 (anti-inflammatory) [59].

These plant extracts can act as modulators of skin immunity processes and possess immunologically as well as dermatologically active compounds. They exert a beneficial effect on skin physiology and are considered promising for treating various skin diseases.

## 4. Bacterial Skin Disease

Skin and soft tissue infections related to bacterial invasion are very common phenomena in emergency care settings. One of the leading risk factors is injury, resulting in disruption of the integrity of the skin [60]. The wounds are divided into acute and chronic forms caused by external damage agents or endogenous mechanisms, respectively. Acute are categorized as surgical, bites, burns, cuts and abrasions, lacerations, crush, and gunshot injuries, whereas chronic include ulcers and pressure sores. Skin damage results in colonization by microorganisms and increases the risk of infection [61], including gas gangrene and tetanus, and may, in turn, lead to even bone infection, and death [62]. Wounds can be contaminated by aerobic and anaerobic microorganisms from the surrounding skin, the environment, and the endogenous sources. The most common bacteria skin pathogens isolated from wounds are *Staphylococcus aureus*, *Pseudomonas aeruginosa*, *Streptococcus* spp., *Escherichia coli*, Gram-negative anaerobes including *Prevotella*, *Porphyromonas* spp., *Bacteroides*, *Fusobacterium* spp., *Peptostreptococcus* spp., and *Clostridium* spp. [61]. Classic signs of wound infection include redness, heat, pain, and swelling of surrounding tissue followed by exudate, poor healing, contact bleeding, epithelial bridging that provides the presence tissue breakdown, unhealthy granulation tissue, and systemic illness [63].

Numerous plant extracts possess antibacterial activities and are effective agents in the treatment of infectious skin diseases, including different types of wounds. Many pathogenic bacterial strains are resistant to antimicrobials, so there is a need to search for new sources of antibacterial agents for external use, where natural products may offer a solution to this problem. Selected plants utilized for bacterial skin ailment management are presented in Table 1.

There are various mechanisms of antibacterial action of plant extracts. The antibacterial activities of numerous phytochemicals are related to the synthesis or function of key components of bacteria and interfere in antibacterial resistance mechanisms. The first one covered disruption of cell wall and protein biosynthesis, inhibition of nucleic acid synthesis, and destruction of cell membranes, whereas the second one overlapped efflux pump interruption, modification of porins and antibiotics, destroying antibacterial agents, and altered targets [64].

The first aspect of antibacterial action is connected with blocked synthesis or function of important bacteria components. *Phyllanthus emblica* and *Lycium shawii* seed extracts were suggested to have remarkable activity against *S. aureus*: after exposure, the entire cells were fully lysed, disrupted or exploded after 3 and 6 h, respectively [65].

Data indicate that plant extracts from *Hibiscus sabdariffa* flowers, *Rosmarinus officinalis* leaves, *Syzygium aromaticum* flowers, and *Thymus vulgaris* leaves may significantly affect the cell membrane of both Gram-positive and Gram-negative bacteria, including *S. aureus*. A decrease in internal pH and membrane hyperpolarization are demonstrated after extract treatment and are indicators of bacterial cell membrane damage [66]. Another antimicrobial agent targeting bacterial cell membranes are flavonoids including glabrol, licochalcone A, licochalcone C, and licochalcone E from licorice, the root and rhizome of *Glycyrrhiza* spp. They exhibit activity against methicillin-resistant *S. aureus* (MRSA), probably via binding to peptidoglycan, phosphatidylglycerol, and cardiolipin, dissipating proton move force, and increasing membrane permeability [67].

Inhibition of amino acid synthesis via inactivation of ribosomes is another target of plants that exert antibacterial activities. It was investigated that aspidinol extracted from *Dryopteris fragrans* exert that effects MRSA [68].

Botanicals as sources of bioactive compounds possess the ability to modulate bacterial multidrug resistance. Alkaloid extracts from *Callistemon citrinus* and *Vernonia adoensis* leaves have shown activity against *P. aeruginosa* via efflux pump inhibition. Various extracts demonstrate synergy with antibiotics [69,70,71,72,73,74] and their bioactive compounds are able to inhibit the expression of bacterial genes, particularly those related to toxin release [75,76,77].

Biofilm is an architectural colony of bacteria forming a complex microbial community. These structures are usually pathogenic, and the data indicate that within all microbial and chronic infections, 65% and 80% are related to biofilm formation, respectively [78]. *P. aeruginosa*, *S. aureus*, and *Enterococcus* spp. are primary pathogens that are responsible for biofilm formation on chronic wounds [79]. Many plant extracts are able prevent or even inhibit formation of bacteria biofilm-positive strains and are important agents for managing biofilm-related diseases [80,81,82,83,84].

Particular plant extracts also take part in the modulation of host inflammation processes. On the one hand, *Withania somnifera* upregulates mRNA expression of pro-inflammatory interleukin-7 (IL-7) [85]; however, it also decreases NFκB transcription factor, which induces the expression of several pro-inflammatory genes [86] and suppresses the intercellular TNF pro-inflammatory cytokine [87]. *Warbugia ugandensis*, *Prunus africana*, and *Plectrunthus barbatus* extracts downregulate IL-7 mRNA expression. This antimicrobial activity may be a consequence of immunopotentiation effects (*W. somnifera*) but the other three act as immunomodulators [85].

Numerous secondary metabolites contained in plants such as flavonoids [88], terpenoids [89], tannins [90], and alkaloids [91] display a wide range of biological activity, including antimicrobial properties. In most cases, cytotoxicity towards mammalian cells of tested plant extracts or plant-extracted molecules was negligible.

## 5. Viral Skin Disease

Viruses are responsible for numerous infections of medical importance as well as a wide range of skin diseases. Among viral skin diseases, herpes simplex viruses (HSV) are ubiquitous. Worldwide, about 80 percent of the population under 50 has type 1 or 2 HSV [112]. The typical person to person transmission is via infected oral secretion or sexual activity by HSV-1 and HSV-2, respectively. The common manifestations of HSV are gingivostomatitis, keratitis, and encephalitis, mostly associated with HSV-1; genital and neonatal infections, and increases in HIV acquisition and transmission, predominantly associated with HSV-2 [113]. HSV-1, belonging to the *Alphaherpesvirinae* subfamily of herpesviruses, is responsible for skin-derived signs and symptoms.

Traditional antiviral pharmacotherapy is capable of inhibiting viral attachment and entry into the host cells. Specific agents also block uncoating, gene expression, genome replication, and viral maturation and release. Numerous plant extracts also possess antiviral activity. This chapter focuses on plant activity against HSV viruses (Table 2) and their mechanisms of action.

Bioactive compounds contained in plant extracts are able to inhibit virus particles in almost every step of the life cycle. HSV-1 is an enveloped virus and their capsid is surrounded by protein and phospholipids. One of the first stages is adsorption and entry into the host cells [114,115]. After the viral molecule binds to the appropriate receptor, cell membrane penetration occurs. This stage requires the delivery of the virus’ genome into host cells, which is usually done by fusion of virus and host cell membrane [116]. Numerous plant-derived natural compounds [117,118,119,120,121,122,123,124,125,126,127,128] modulate the mechanisms of virus entry through prevention of attachment and inhibition of penetration mechanisms controlled via receptors, enzymes, and several chemicals. Extract of *Rheum officinale* rhizomes, *Paeonia suffruticosa* roots, *Melia toosendan* fruits, and *Sophora flavescent* roots prevent, inhibit or modulate that step [117]. Phyllanthus *orbicularis* stems and leaves inhibit the binding of the cellular receptor [121], as do *Azardirachta indica* bark [122], *Melissa officinalis* leaves [123], *Cissus repanda* leaves and climbers [124], and *Hemidesmus indicus* root [125] extracts. Viral adsorption inhibitory effect was observed for *Cassia sieberiana* aerial parts and *Guiera senegalensis* aerial part extracts [129]. Moreover, lectin isolated from *Euphorbia* affected the virus entry to the host cells [120]. Data indicate that extracts of *Rheum officinale* rhizomes and *Paeonia suffruticosa* roots additionally enable virus penetration [117]. A similar biological effect is observed for *Rhus aromatica* roots/stems barks [126], *Azardirachta indica* barks [122], *Limonium densiflorum* shoots [127], *Hibiscus* whole plants [128], and *Nepeta nuda* aerial parts [119].

The HSV-1 genome consists of approximately 152 kbp double-stranded linear DNA [130] within the capsid, which encodes seven proteins taking part in synthesis of nucleic acid [131]. It was observed that many plant extracts have the ability to disrupt this process, including *Senna podocarpa* leaves, *Cassia sieberiana* roots, *Guiera senegalensis* aerial parts, *Piliostigma thonningi* aerial parts, *Rhamnus glandulosa* leaves, and *Uvaria chamae* roots [129]. Limited replication is also observed after exposure to Lychee flower extract on rabbit corneal epithelia cells [132]. Data indicated that pearl garlic extract inhibited the replication in about 70% of the acyclovir resistant HSV-1 thymidine kinase mutants [133]. *Limonium brasiliense*, *Psidium guajava*, and *Phyllanthus niruri* also exhibit antiherpetic activity and inhibit virus replication [134]. Suppressor effects on HSV-1 replication related to inhibition of DNA synthesis and gene expression were identified also from *Nelumbo nucifera* seeds extract in treated HeLa cells by disturbing the formation of α-trans-induction factor/C1/Oct-1/GARAT multiprotein/DNA complexes [135].

Some plant compounds also directly interact with virus particles and neutralize them. *Aloe barbadensis* leaves inactivate HSV-1 viruses by disruption of the envelope [136], whereas *Orthosiphon stamineus* leaves, flowers, and whole plants extracts disintegrate HSV-1 structure [137]. Virucidal mechanisms of action are also proposed for *Phyllanthus orbicularis* [138] stem and leaves, *Allium sativum* [139] cloves, *Senna podocarpa* leaves, *Cassia sieberiana* aerial parts, *Guiera senegalensis* aerial parts, *Lippia chevalieri* aerial parts, *Pavetta oblongifolia* steams, aerial parts and roots, *Piliostigma thonningii* aerial parts, *Rhamnus glandulosa* leaves, *Sarcocephalus latifolius* roots, and *Terminalia macroptera* roots extracts [129].

During infections caused by viruses, an important role is played by autophagy. This process is initiated by inactivation of mammalian targets to rapamycin (mTOR), a kinase that takes part in cell proliferation and protein synthesis via activation of ribosomal p70S6 kinase. Lychee flowers extract treatment inactivates mTOR by decreasing its phosphorylation and enables activation of autophagy by expression of the proteins necessary for phagophore nucleation and elongation as well as transport and maturation of the autophagosome; during HSV-1 infection without extract treatment, the phosphorylated level of mTOR increased [132].

Numerous particular plant metabolites possess antiviral properties: alkaloids, coumarins, flavonoids, lignans, miscellaneous compounds, monoterpenoids, diterpenoids and sesquiterpenoids, phenolic, phenylpropanoids, quinones, tannins, thiophenes and polyacetylenes, and triterpenoids [140]. The results demonstrate that the extracts prepared from plants possess potent phytochemical compounds that were mainly noncytotoxic to mammalian cells.

## 6. Fungal Skin Disease

Dermatophytes and yeast are two classes of fungal skin pathogens.

The dermatophytes invade keratinized tissues including skin, hair, and nails and mainly penetrate the nonliving cornified layers of the skin. The etiological agents are anamorphic fungi from *Hyphomycetes* class, including the genera *Epidermophyton*, *Microsporum*, and *Trichophyton*. The host reaction to dermatophyte infections is closely related to the strains of fungi, location, and environmental factors; these may be mild or severe, resulting in disease such as tinea capitis, tinea pedis, and onychomycosis [178,179]. Dermatophytosis risk factors include poor hygiene, administration of immunosuppressive drugs, and diabetes mellitus [180].

*Candida* species are yeast skin pathogens; *Candida albicans* is a dermatologically important species responsible for the largest number of skin infections related to skin thickening, hyperkeratosis, and erythema. Skin colonization by this strain is related to atopic dermatitis and psoriasis [181]. It is a commensal microorganism that is present in oral, conjunctival, likewise, gastrointestinal and genitourinary tracts in a normal human body, while infections occur in debilitated and immunocompromised patients. *Candida albicans* infections usually develop on mucous membranes but in some cases, can spread into the bloodstream and then, to the vital organs and be life threating. Candidiasis risk factors are as follows: malignancies, immunosuppressive disease, administration of some pharmaceuticals including broad-spectrum antibiotics, parenteral nutrition, medical management including surgery, chemotherapy, and transplantation, and in addition, central venous catheters and internal prosthetics [182,183].

The genus *Malassezia* is a yeast that resides on normal skin and is an important component of the mycobiome. It is unable to synthetize fatty acids. *Malessezia* is involved in the pathology of numerous skin disease including pityriasis versicolor, seborrheic dermatitis, folliculitis, and atopic dermatitis. The genus contains 17 species. Among the species, the most prevalent types related to both healthy and diseased skin are *M. restricta*, *M. globosa*, and *M. sympodialis*, whereas *M. furfur* is also common but strictly related to skin disorders [184,185].

Many traditionally used plants (Table 3) can be useful for relieving fungal infections and exhibit low side effects; its inhibitory effects on fungi cultures are comparable to traditional drugs. Plant-derived antifungal agents can act on different targets.

The antifungal activity of herbal compounds seems to be related to the modulation of the immune system. Leaves and tender branches of *Larrea divaricate* extract activate murine macrophages, which can increase its ability to phagocytose *C. albicans*. Extracts in the presence of fungus cells potentiate the superoxide anion and nitric oxide (NO) production [186]. A similar effect was observed for *Phyllostachys bambusoides* leaf extract, where elevated NO production results in improvement of macrophage phagocytose action and enables effective eradication of *C. albicans* from Balb/c mice as a consequence of elevated levels of IFN-γ, IL-2, and IL-4 release [187]. *Allium sativum* clove extract improved the removal of *Sporothrix schenckii*, the causal agent of sporotrichosis, common subcutaneous mycosis. Regular consumption increases the level of pro-inflammatory cytokines such as IL-1β, IL-12 in infected Swiss mice, and anti-inflammatory cytokine IL-10 in healthy animals. Excessive inflammation may be a natural approach to treating fungal infection [188].

Plant-derived compounds may also exert an influence on fungal grown and induce programmed cell death. *T. rubrum* and *T. mentagrophytes* treated with *Allium sativum* cloves extract show degradation of hypha components, including cell wall, membrane, and cytoplasm [189]; treatment with *Panax notoginseng* roots extract, particularly heat-transformed saponins, might damage the cell membrane of dermatophytes followed by decrease in membrane potential [190]. The proposed mechanism of action of *Cassia fistula* seeds extract on *C. albicans* revealed that the extract exerts an effect on cell membrane and initiate their disruption, resulting in damage to yeast cells [191]. *Nyctanthes arbor tristis* leaves extract affected *M. restricta* cell membrane [192]. *Lafoensia pacari* stem-bark extract and their main component ellagic acid probably exert an impact on the *Candida* spp. cell wall. Ellagic acids were identified as antifungal agents by acting on ergosterol [193]. Moreover, natural compounds can directly interact with fungal cells, collapsing them via overproduction of reactive oxygen species (ROS). Extract from the roots of *Scutellaria baicalensis* contains two main antifungal components: baicalein and wogonin. Their action is connected with an excessive level of ROS in fungal cells, which leads to induction of apoptosis [194]. Baicalein possesses potent antifungal activity against *T. rubrum*, *T. mentagrophytes*, *Aspergillus fumigatus*, and *C. albicans*, whereas wogonin does not demonstrate activity against *C. albicans*.

Numerous metabolites contained in plants such as polyphenols, terpenes, and nitrogenous compounds display a wide range of biological activity including antifungal properties [195].

## 7. Conclusions

Skin diseases occur in people of all ages, from newborns to the elderly, and are typically caused by bacteria, viruses, and fungi. The most popular skin invaders are linked to wound infection, herpes, and tinea. In addition to diagnosis, the prevention and treatment of diseases should also focus on enhancement of life quality. In this context, new strategies and novel experimental approaches for the relief of skin problems of people suffering from nonfatal disability are desired. The first line of defense against pathogens is the skin, the largest organ in the human body. Keratinocytes, Langerhans cells, mast cells, and T cells are the important immune skin cells involved in innate and adaptive reactions. The second line of defense should constitute pharmacotherapy.

Plant compounds may represent an alternative to traditional pharmaceuticals. Plant extracts can act as immunomodulators of the cutaneous response and possess both anti-inflammatory and pro-inflammatory properties. Moreover, the plant kingdom is a rich source of active ingredients that exert antibacterial, antiviral, and antifungal effects, thanks to their biologically active chemicals. The potential mechanism of action is varied and depends on the main compounds found in the plant extract, but to a large extent, the therapeutic effect focuses on the synergistic action of these compounds.

Plant extracts have been found to have inhibitory effects on the growth of bacteria, viruses, and fungi in in vitro/in vivo studies. Their activities are frequently related to the blockage of the synthesis or function of key components of particular organisms. Moreover, natural compounds may interfere with bacterial resistance mechanisms, disrupting viral attachment and entry into the host. For centuries, plants were widely used as medications.

Although more than 50% of plant species are used to treat skin diseases, many of them can be destroyed by human activities such as habitat destruction, deforestation or urbanization. Moreover, it should also be remembered, however, that plant extracts may cause skin allergies or enhance its action in some people, but in this paper, they have shown promising results.

This review helps the researchers working on skin problems to screen out the efficient or to find out new approaches in reported plants, which may be a step ahead in the drug discovery process.

## Figures and Tables

**Table 1:** Antibacterial effect of plant extracts.

Name of The Species/Family	Part of the Plant	Type of Extract	Active Compounds/ Class of Compounds	Pathogens	Ref.
*Chromolaena odorata* L./Asteraceae	leaf	methanolic	-	*Pseudomonas aeruginosa*	[92]
*Chromolaena odorata* L./Asteraceae	leaf, stem, roots	ethanolic, methanolic and hexane	phenolics and flavonoids	*Bacillus cereus*, *Enterococcus faecalis*, *Staphylococcus epidermidis*, *Staphylococcus aureus*, *Streptococcus pyogenes Propionibacterium acnes, Proteus vulgaris*	[93]
*Brachylaena elliptica* (Thunb.) DC./Asteraceae, *Brachylaen ilicifolia* (Thunb.) DC./Asteraceae	leaf	ethanolic	tannins, phenols, flavonoids, flavonols, proanthocyanidins, alkaloids, saponins	*Pseudomonas aeruginosa, Proteus vulgaris, Proteus mirabilis, Staphylococcus aureus* *Streptococcus pyogenes*	[94]
*Swietenia macrophylla* King./Meliaceae	seed	methanolic	21 compounds with oleic acid and linoleic acid were the main components	*Staphylococcus* *aureus, Bacillus cereus, Bacillus subtilis, Proteus mirabilis, Yersinia spp., Escherichia coli, Klebsiella* *pneumoniae, Shigella boydii and Acinobacter anitratus*	[95]
*Pimpinella anisum* L./Apiaceae	whole plant	methanolic	phytoestrogen, flavonoids	*Staphylococcus aureus*, *Pseudomonas aeruginosa*	[96]
*Croton lobatus* L./Euphorbiaceae	leaf	water	tannins, triterpenoids, flavonoids, saponins	*Staphylococcus aureus, Streptococcus spp*; *Pseudomonas aeruginosa, Proteus vulgaris*; *Escherichia coli*	[97]
*Citrus aurantifolia* Hort. ex Tanaka/Rutaceae	lime peel	ethanolic	*-*	*Staphylococcus aureus*	[98]
*Elaeis guineensis* Jacq./Arecaceae	root	n-hexane, chloroform, ethyl acetate and butanol	tannins, saponins, steroids and flavonoids	*Staphylococcus aureus, Pseudomonas aeruginosa*	[99]
*Alhagi maurorum* Medik./Fabaceae	leaf, stem	water	-	Nineteen *Pseudomonas aeruginosa* strains	[100]
*Zizania latifolia (Griseb.)* Turcz. ex Stapf./Poaceae	aerial parts	water	-	*Staphylococcus aureus*	[101]
*Opuntia ficus-indica* Mill./Cactaceae	flower	methanolic	monosaccharides	*Escherichia coli, Staphylococcus aureus, Bacillus subtilis, Pseudomonas aeruginosa and Listeria monocytogenes*	[102]
*Alkanna strigose Boiss&Hohen./*Boraginaceae	root	hexane	Alkanin, shikonin	*Staphylococcus aureus, Pseudomonas-aeruginosa, Escherichia coli,* *Bacillus subtilis*	[103]
*Salvia kronenburgii* Rech. f./Lamiaceae, *Salvia euphratica* Montbret, Aucher and Rech. f. var./Lamiaceae	aerial part	ethanolic	phenolics and flavonoids	*Staphylococcus aureus, Bacillus subtilis, Escherichia coli, Acinetobacter baumannii, Aeromonas hydrophila, Mycobacterium tuberculosis*	[104]
*Juglans regia* L./Inner Stratum of Oak Fruit (Jaft)/Juglandaceae	fruit, stem bark	ethanolic	*-*	*Staphylococcus aureus*	[105]
*Garcinia mangostana* L./Clusiaceae	pericarp	methanolic	α-mangostin	*Staphylococcus aureus*	[106]
*Ficus thonningii* Blume./Moraceae	leaf	ethanolic	alkaloids, phlobatannins, steroids, saponins	*Escherichia coli*, *Klebsiella pneumoniae*, *Proteus mirabilis*, *Pseudomonas aeruginosa*, *and Staphylococcus aureus*	[107]
*Balanites aegyptiaca* L. Delile./Zygophyllaceae	bark	water:ethanolic	phenolics	*Pseudomonas aeruginosa* and *Staphylococcus aureus*	[108]
*Myrciaria cauliflora* (Mart.) O. Berg./Myrtaceae	crude	methanolic	-	*Staphylococcus aureus*	[109]
*Acacia ehrenbergiana* Hayne (Salam)./Fabaceae	Stem, bark	methanolic	-	*Pseudomonas aeruginosa*	[110]
*Acacia nilotica* Lam./Fabaceae	seed	methanolic extract/emulsifying ointment	-	*Staphylococcus aureus, Pseudomonas aeruginosa, Escherichia coli, Klebsiella pneumonia, Streptococcus pyrogens and Salmonella typhi*	[111]

**Table 2:** Antiviral effect of plant extracts.

Name of the Species/Family	Part of the Plant	Type of Extract	Active Compounds/ Class of Compounds	*Pathogens*	Ref.
*Euphorbia cooperi* N.E.Br. ex A.Berger/Euphorbiaceae	leaf, flower	water, methanolic	polyphenols	*Herpes Simplex Virus type 1 (HSV-1)*	[141]
*Euphorbia cooperi* L./Euphorbiaceae, *Odina wodier* Roxb./Anacardiaceae, *Moringa oleifera* Lam./Moringaceae	Fruit, bark, leaf, flower	methanolic	-	*HSV-1 and Herpes Simplex Virus type 2 (HSV-2)*	[142]
*Cornus canadensis* L./Cornaceae	leaf	water:ethanolic	hydrolysable tannins	*Herpes Simplex Virus type 1 (HSV-1)*	[143]
*Eleusine indica* L. Gaertn./Poaceae	whole plant	methanol, hexane	tannins, alkaloids, steroids	*Herpes Simplex Virus type 1 (HSV-1)*	[144]
*Pistacia lentiscus* L./Anacardiaceae, *Peganum harmala* L./Nitrariaceae	seed, stem, fruit, flower	methanolic, dichloromethane, acetate, hexane, ethanolic	-	*Herpes Simplex Virus type 2 (HSV-2)*	[145]
*Orthosiphon stamineus* Benth./Lamiaceae	leaf	water, methanolic, ethanolic	alkaloids, flavonoids, steroids, terpenoids, anthraquinone, saponins	*Herpes Simplex Virus type 1 (HSV-1)*	[146]
*Arctium lappa* L./Asteraceae	fruit	water:ethanolic	dibenzylbutyrolactone lignans, such as arctiin and arctigenin	*Herpes Simplex Virus type 1 (HSV-1)*	[147]
*Asplenium nidus* L./Aspleniceae	leaf	water	alkaloids, flavonoids, terpenoids, anthraquinones	*Herpes Simplex Virus type 1 (* *HSV-1)*	[148]
*Capsicum annuum* L./Solanaceae	whole fruits	methanolic	phenolic, flavonoids	*Herpes Simplex Virus type 1 (HSV-1) and Herpes Simplex Virus type 2 (HSV-2)*	[149]
*Chelidonium majus* L./Papaveraceae	herbs	hexane, water	-	*Herpes Simplex Virus type 1 (HSV-1)*	[150]
*Chrysanthemum cinerariaefolium* (Trevir.) Sch. Bip./Asteraceae	flowers	water, methanolic	alkaloids, flavonoids, phenols, saponins, tannins and terpenoids	*Herpes Simplex Virus type 1 (HSV-1)*	[151]
*Cinnamon* Scheffer./Lauraceae	cinnamon tree	water:methanolic	-	*Herpes Simplex Virus type 1 (HSV* *-1)*	[152]
*Phellodendron* *amurense* Rupr./Rutaceae	bark	water	berberine	*A virus (PR8), Vesicular Stomatitis Virus (VSV), Herpes Simplex Virus (HSV), Enterovirus-71 (EV-71)*	[153]
*Durian, Durio zibethinus* Murray./Malvaceae	seed	water, ethanolic	-	*Herpes Simplex Virus type 2 (HSV-2)*	[154]
*Euphorbia spinidens* Bornm./Euphorbiaceae	aerial part	methanolic	phenolics, flavonoids	*Herpes Simplex Virus type 1 (HSV-1)*	[155]
*Ficus religiosa* L./Moraceae	bark, leaf	water, methanolic, ethyl acetate, chloroform	-	*Herpes Simplex Virus type 2 (HSV-2)*	[156]
*Fridericia formosa* (Bureau) L. G. Lohmann./Bignoniaceae	leaves, stems, and fruits	ethanolic	C-glucosylxanthones mangiferin, 2-O-trans-caffeoylmangiferin, 2-O-trans-coumaroylmangiferin, 2- O-trans-cinnamoylmangiferin, flavonoid chrysin	*Herpes Simplex Virus type 1 (* *HSV-1), murine encephalomyocarditis virus (EMCV)*	[157]
*Graptopetalum paraguayense* E. Walther./Crassulaceae	leaves	water:methanolic	-	*Herpes Simplex Virus type (HSV)*	[158]
*Houttuynia cordata* Thunb./Saururaceae	aerial part	water, ethanolic	-	*Herpes Simplex Virus type 1 (HSV-1) and Herpes Simplex Virus type 2 (HSV-2)*	[159]
*Ixeris Sonchifolia* (Bae.) Hance./Asteracae	whole grass or root	-	flavonoids	*Herpes Simplex Virus type 1 (HSV-1)*	[160]
*Jatropha multifida* L./Euphorbiaceae	root	n-hexane, ethanolic and methanolic	lathyrane-type diterpenes	*Herpes Simplex Virus type 1 (HSV-1)*	[161]
*Glycyrrhiza glabra* L./Fabaceae	root	water, alkaline	flavonoids and chalcone derivatives (liquiritin apioside, liquiritigenin 7-apiosylglucoside, liquiritin, neoliquiritin, liquiritigenin, isoliquiritin apioside, licurazid, isoliquiritin, neoisoliquiritin, isoliquiritigenin)	*Herpes Simplex Virus type 1 (HSV-1) and HIV virus*	[162]
*Morus alba* L./Moraceae	leaf	-	phenolics, flavonoids	*Herpes Simplex Virus type 1 (HSV-1) and Herpes Simplex Virus type 2 (HSV-2)*	[163]
*Peganum harmala* L./ Nitrariaceae	seed, stem, leaf, and flower	hexane, dichloromethane, ethyl acetate, methanolic, ethanolic	alkaloids	*Herpes Simplex Virus type 2 (HSV-2)*	[164]
*Phaleria macrocarpa* (Scheff.) Boerl./Thymelaeaceae	fruit	water	steroids, tannins, flavones aglycones, saponins, terpenoids, alkaloids	*Herpes Simplex Virus type 1 (HSV-1)*	[165]
*Pistacia vera* L./Anacardiaceae	pistachio kernels	-	polyphenols	*Herpes Simplex Virus type 1 (HSV-1)*	[166]
*Punica granatum* L./Lythraceae	fruit peel	water, water:ethanolic, ethanolic	ellagitannin, gallotannin	*Herpes Simplex Virus type 2 (HSV-2)*	[167]
*Rhinacanthus nasutus* L. Kurz./Acanthaceae	stem	ethanolic, ethylacetate, methanolic, dichloromethane, acetone and hexane	-	*Herpes Simplex Virus type 2 (HSV-2)*	[168]
*Robinia pseudoacacia cv.* Idaho./Fabaceae	flower	ethanolic	flavonoids	*Human enterovirus 71 virus (EV-71) and Herpes Simplex Virus type 1 (HSV-1)*	[169]
*Solanum melongena* L./Solanaceae	berry peel	ethanolic	phenolics, flavonoids	*Herpes Simplex Virus type 1 (HSV-1)*	[170]
*Strychnos pseudoquina* A. St. Hil/Loganiaceae	stem bark	ethyl acetate	flavonoids	*Herpes Simplex Virus type 1 (HSV-1) and Herpes Simplex Virus type 2 (HSV-2)*	[171]
*Tanacetum parthenium* L. Sch.Bip./Asteraceae	aerial part	water:ethanolic	phenolic acids (chlorogenic acids), sesquiterpene lactones (parthenolide)	*Herpes Simplex Virus type 1 (HSV-1)*	[172]
*Terminalia chebula* Retz./Combretaceae	fruit	ethanolic	chebulagic acid, chebulinic	*Herpes Simplex Virus type 2 (HSV-2)*	[173]
*Veronica persica* Poir./Plantaginaceae	stem, leaf, flower	methanolic	-	*Herpes Simplex Virus type 1 (HSV-1) and Herpes Simplex Virus type 2 (HSV-2)*	[174]
*Polygonum minus Huds./*Polygonaceae	leaf, stem	methanolic	-	*Herpes Simplex Virus type 1 (HSV-1)*	[175]
*Lamium album* L./Lamiaceae	aerial part	water, ethanolic	-	*Herpes Simplex Virus type 1 (HSV-1) and Herpes Simplex Virus type 2 (HSV-2)*	[176]
*Cuminum cyminum* L./Apiaceae	seed	methanolic	phenolics, flavonoids	*Herpes Simplex Virus type 1 (HSV-1) and Herpes Simplex Virus type 2 (HSV-2)*	[177]

**Table 3:** Antifungal effect of plant extracts.

Name of the Species/Family	Part of the Plant	Extract/Active Compounds	Active Compounds/ Class of Compounds	*Pathogens*	Ref.
*Dryopteris fragrans* L. Schott./Dryopteridaceae	whole plant	ethanolic	Fourteen derivatives of phloroglucinol	*62 isolates of dermatophytes (such as Trichophyton mentagrophytes or Trichophyton rubrum*	[196]
*Vismia rubescens Oliv./*Hypericaceae	stem bark	methanolic	1,4,8-trihydroxyxanthone, 1,7-dihydroxyxanthone, physcion, friedelin and friedelanol	*Candida albicans, Candida tropicalis, Candida parapsilosis and Cryptococcus neoformans*	[197]
*Saraca indica* L. *(Ashoka tree)/*Fabaceae, *Eucalyptus citriodora* L./Myrtaceae	leaf	methanolic	-	*Microsporum nanum*, *Microsporum gypseum, M. canis*, *Aspergillus niger*, *Rhizopus, Alternaria, Candida, Penicillium*	[198]
*Ageratum conyzoides L./*Asteraceae	leaf	methanolic	flavonoids, phenolics and tannins	*Microsporum gypseum, Trichophyton violaceum and Epidermophiton floccosum*	[199]
*Croton tiglium* L./Euphorbiaceae	stem, leaf, seed	ethanolic	-	*Trichophyton mentagrophytes*, *Trichophyton rubrum*, *andEpidermophyton floccosum*	[200]
*Allium sativum* L./Amaryllidaceae	fresh bulbs of garlic	water, ethanolic and methanolic	-	*Trichophyton mentagrophytes, Trichophyton rubrum, Microsporum gypseum, Trichophyton verrucosum and Epidermophyton floccosum*	[201]
*Cola nitida* (Vent.) Schott and Endl./Malvaceae	stem bark	ethanolic	-	*Trichophyton rubrum* *,* *Trichophyton tonsurans*	[202]
*Cleome gynandra* L./Cleomaceae	aerial part	water, methanolic	alkaloids, flavonoids, tannins, terpenoids, steroids	*Trichophyton rubrum, Microsporum canis* *and* *Trichophyton mentagrophytes*	[203]
*Solanum nigrum L./*Solanaceae	fruit	methanolic	-	*Microsporum canis*	[204]
*Peganum harmala* L./Nitrariaceae, *Echinophora platyoba* DC./Umbelliferae, *Rosmarinus officinalis* L./Lamiaceae and *Heracleum persicum* L./Apiaceae	whole plant	methanolic	-	*Candida albicans*	[205]
*Cocos nucifera* L./Arecaceae	fruit	methanolic	phenolic compounds	*Aspergillus niger, Microsporum canis,* *Microsporum gypseum, Aspergillus flavus, Trichophyton rubrum, Aspergillus fumigatus* *and* *T* *richophyton* *vercossum* *. Mycosis clinically isolated from* *Tinea corporis*	[206]
*Grewia asiatica* L./ Malvaceae	leaves	acetonic	phenolics, flavonoids, alkaloids, tannins, terpenoids, saponins	*Aspergillus spp., Candida spp., Microsporum spp., Trichophyton* spp.	[207]
*Nectaroscordum tripedale* (Trautv.) Traub./Alliaceae	aerial parts	methanolic	24 compounds with the main compounds such as: decadienal hexadecanoic acid, heptadecane	*Trichophyton mentagrophytes, Microsporum canis, Microsporum gypseum*	[208]
*Dittrichia viscosa* (L.) Greuter./Asteraceae	leaves	methanolic, ethanolic, and butanolic	tannins, phenols, flavonoids	*Malasseziaspp.,Aspergillus* spp.	[209]
*Cinnamomum zeylanicum* Blume./Lauraceae, *Zingiber officinale* Rosc./Zingiberaceae, *Heracleum persicum* Boiss./Apiaceae, *Elettaria cardamomum* L./*Zingiberaceae*, *Salvia officinalis* L./Lamiaceae, *Calendula officinalis* L./Astereceae, *Myrtus* *communis* L./Myrtaceae, *Mentha spicata* L./Lamiaceae	flower, bark, seed, leaf, aerial part, rhizome	methanolic	Flavonoids, tannins, phenolics, alkaloids	*Trichophyton mentagrophytes, Trichophyton interdigitale,Microsporum* *canis*, *andMicrosporum gypseum*	[210]
*Amygdalus eburnean* Spach./Rosaceae	shell root	methanolic water	-	*Trichophyton mentagrophytes and Trichophyton interdigitale*	[211]
*Barleria grandiflora* Dalz./Acanthaceae	leaf	water	-	*Aspergillus fumigatus*	[212]
*Punica granatum* L./Lythraceae, *Illicium verum* Hook./Schisandraceae, *Nyctanthes arbor-tristis* L./Oleaceae, *Thespesia populnea* L./Malvaceae, *Piper betle* L./Piperaceae	fruit rind, fruit, leaf	chloroform, hexane, water, methanolic	-	*Malassezia furfur*	[213]
*Cola nitida* Schott and Endl./Malvaceae	leaf and seed	methanolic	anthraquinones, tannins, saponins, alkaloids and cardenolides	*Candida valida, Candida glabrata, Candida tropicalis, Candida albicans, Candida krusei, Trichophyton interdigitalis, Trichophyton tonsurans, Epidermytophyton rubrum Trichophyton rubrum*	[214]
*Leucas aspera* (L.) R. Br./Lamiaceae, *Leucas zeylanica* (L.) W.T.Aiton./Lamiaceae	leaf	methanolic	-	*Aspergillus flavus, Candida albicans, Candida tropicalis, Epidermophyton floccosum, Microsporum nanum, Penicillium, Trichophyton mentagrophytes*	[215]
*Commiphora molmol* Engl./Burseraceae	myrrh	ethanolic	Furanoeudesma 1,3-diene and menthofuran, 2-tert-butyl-1,4-naphthoquinone, benzenemethanol,3-methoxy-a-phenyl, curzerene	*Trichophyton rubrum* *,* *Trichophyton mentagrophytes* *,* *Microsporum canis* *,* *M. gypseum,* *and* *Trichophyton verrucosum*	[216]
*Drimia sanguinea* (Schinz) Jessop./Asparagaceae, *Elephantorrhiza elephantine* (Burch.) Skeels./Fabaceae, *Helichrysum paronychioides* Mill./Asteraceae, *Senecio longiflorus* (DC.) Sch.Bip./Asteraceae	whole plant	methanolic	phenolics and flavonoids	*Candida glabrata, Candida krusei, Trichophyton rubrum and Trichophyton tonsurans*	[217]
*Ocimum gratissimum* L./Lamiaceae, *Vernonia amygdalina* Delile./Asteraceae	leaf	ethanolic	-	*Microsporum* spp., *Trichophyton* spp., *Aspergillus* spp., and *Penicillium* spp.	[218]
*Trigonellafoenum-graecum* L./Fabaceae	leaf	water	Flavonoids, alkaloids, saponins, phenolics	*Malassezia furfur, Aspergillus niger, Candida albicans*	[219]
*Albizia saman* (Jacq.) Merr/Fabaceae	leaf	methanolic	-	*Malassezia* spp.	[220]
*Azadirachta indica* A. Juss./Meliaceae	bark neem	diethyl ether, chloroform, acetone, and ethanolic	alkaloids, flavonoids, tannins, phenolics, terpenoids (nimbin, nimbolinin and nimbidin) and steroids (nimbidol)	*Malassezia globosa and Malassezia restricta*	[221]
*Citrus aurantifolia* Hort. ex Tanaka/Rutaceae, *Curcuma domestica* L./*Zingiberaceae*, *Trigonella foenumgraceum* L./Fabaceae, *Cassia alata* (L.) Roxb./Fabaceae, *Azadirachta indica* A. Juss./Meliaceae	leaf, rhizome, seed, exocarp,	methanolic	-	*Malassezia* spp.	[222]
*Salacia senegalensis* (Lam.) DC/Celastraceae	leaf	aqueous-ethanolic	flavonoids	*Trichophyton rubrum, Epidermophyton floccosum*	[223]
*Ipomoea aquatic* Forsk./Convolvulaceae	apical bud and flower	ethanolic	-	*Tricophyton rubrum, Epidermophyton floccosum, Microsporum gypseum, Malassezia furfur, Malassezia globosa*	[224]
*Betula cylindrostachya* Lindl. ex Wall/Betulaceae	leaf	methanolic, water- methanol, chloroform, ethyl acetate and *n*-butanol	1-heptadecanol, behenyl alcohol, 1-tricosanol, 1-nonacosanol and lignoceric acid	*Malassezia furfur*	[225]
*Ficus sycomorus* L./Moraceae	root, stem-bark, leaf, fruit	methanolic	flavonoids	*Trichophyton mentagrophytes var. erinacei, Malassezia audouinii and Microsporum gypsum, Fennellia nivea, Choaenophora cucurbitarum, Aspergillus carneus and Aspergillus fumigatus*	[226]
*Alpinia nigra* (Gaertn.) B.L.Burtt/Zingiberaceae	rhizome	dichloromethane	flavonoids	*Malassezia furfur and Microsporum gypseum*	[227]
*Plumbago indica* L./Plumbaginaceae	root	acetone	-	*Propionibacterium acnes, Staphylococcus epidermidis, Malassezia furfur*	[228]
*Gmelina asiatica* L./Lamiaceae, *Ipomoea digitate* Jacq./Convolvulaceae	aerial part, tuber	chloroform, ethyl acetate, and methanolic	glycosides, flavonoids, phenolics, tannins, phytosterols, triterpenoids, saponins, alkaloids,	*Malassezia furfur* *,* *Propionibacterium* *acnes* *,* *Corynebacterium diphtheriae*	[229]
*Senna macranthera* DC.ex Colladon/Fabaceae	flower	ethanolic	flavan-3-ol, flavone, glycosylated flavonols, proanthocyanidin	*Candida albicans*	[230]
